# Poly[bis­[μ_4_-*N*-(2-hydroxy­imino­propion­yl)-*N*′-(2-oxidoimino­propion­yl)propane-1,3-diaminato]dimethano­lcalciumdicopper(II)]

**DOI:** 10.1107/S1600536809033467

**Published:** 2009-08-29

**Authors:** Valentina A. Kalibabchuk, Natalia I. Usenko, Irina A. Golenya, Turganbay S. Iskenderov, Matti Haukka

**Affiliations:** a Department of General Chemistry, O. O. Bohomolets National Medical University, Shevchenko Blvd. 13, 01601 Kiev, Ukraine; b Department of Chemistry, Kiev National Taras Shevchenko University, Volodymyrska Street 64, 01033 Kiev, Ukraine; c Department of Chemistry, Karakalpakian University, Universitet Keshesi 1, 742012 Nukus, Uzbekistan; d Department of Chemistry, University of Joensuu, PO Box 111, 80101, Joensuu, Finland

## Abstract

In the title compound, [CaCu_2_(C_9_H_13_N_4_O_4_)_2_(CH_3_OH)_2_]_*n*_, the Ca^II^ atom lies on an inversion center and is situated in a moderately distorted octa­hedral environment. The Cu^II^ atom is in a distorted square-pyramidal geometry, defined by four N atoms belonging to the amide and oxime groups of the triply deprotonated residue of *N*,*N*′-bis­(2-hydroxy­imino­propano­yl)propane-1,3-diamine (H_4_pap) and one oxime O atom from a neighboring Hpap ligand at the apical site, forming a dimeric [Cu_2_(Hpap)_2_]^2−^ unit. Each dimeric unit connects four Ca atoms and each Ca atom links four [Cu_2_(Hpap)_2_]^2−^ units through Ca—O(amide) bonds, leading to a three-dimensional framework. The crystal structure involves intra- and inter­molecular O—H⋯O hydrogen bonds.

## Related literature

For the coordination chemistry of tetra­dentate oxime-and-amide open-chain ligands, see: Duda *et al.* (1997 ▶); Fritsky *et al.* (1999 ▶). For oximes as efficient metal chelators, see: Gumienna-Kontecka *et al.* (2000 ▶); Onindo *et al.* (1995 ▶); Sliva *et al.* (1997*a* ▶,*b* ▶). For the use of oximes in stabilizing high oxidation states of metal ions, see: Fritsky *et al.* (1998 ▶, 2006 ▶). For related structures, see: Kanderal *et al.* (2005 ▶); Fritsky (1999 ▶); Fritsky *et al.* (2000 ▶); Mokhir *et al.* (2002 ▶); Moroz *et al.* (2008 ▶); Wörl *et al.* (2005 ▶).
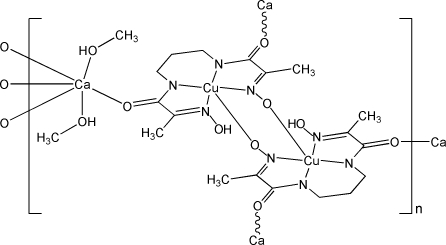


## Experimental

### 

#### Crystal data

[CaCu_2_(C_9_H_13_N_4_O_4_)_2_(CH_4_O)_2_]
*M* *_r_* = 713.71
Monoclinic, 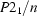

*a* = 10.0554 (4) Å
*b* = 8.7794 (3) Å
*c* = 15.4465 (7) Å
β = 97.882 (2)°
*V* = 1350.74 (9) Å^3^
*Z* = 2
Mo *K*α radiation
μ = 1.83 mm^−1^
*T* = 120 K
0.28 × 0.24 × 0.13 mm

#### Data collection

Nonius KappaCCD diffractometer
Absorption correction: multi-scan (*SADABS*; Sheldrick, 1996 ▶) *T*_min_ = 0.622, *T*_max_ = 0.796
8392 measured reflections
3074 independent reflections
2573 reflections with *I* > 2σ(*I*)
*R*_int_ = 0.035

#### Refinement

*R*[*F* ^2^ > 2σ(*F* ^2^)] = 0.030
*wR*(*F* ^2^) = 0.079
*S* = 1.04
3074 reflections
192 parameters
H-atom parameters constrained
Δρ_max_ = 1.11 e Å^−3^
Δρ_min_ = −0.56 e Å^−3^

### 

Data collection: *COLLECT* (Nonius, 1998 ▶); cell refinement: *DENZO*/*SCALEPACK* (Otwinowski & Minor, 1997 ▶); data reduction: *DENZO*/*SCALEPACK*; program(s) used to solve structure: *SIR2004* (Burla *et al.*, 2005 ▶); program(s) used to refine structure: *SHELXL97* (Sheldrick, 2008 ▶); molecular graphics: *ORTEP-3* (Farrugia, 1997 ▶); software used to prepare material for publication: *SHELXL97*.

## Supplementary Material

Crystal structure: contains datablocks global, I. DOI: 10.1107/S1600536809033467/hy2216sup1.cif

Structure factors: contains datablocks I. DOI: 10.1107/S1600536809033467/hy2216Isup2.hkl

Additional supplementary materials:  crystallographic information; 3D view; checkCIF report

## Figures and Tables

**Table 1:** Selected bond lengths (Å)

Cu1—N1	1.9751 (18)
Cu1—N2	1.9469 (18)
Cu1—N3	1.9320 (19)
Cu1—N4	1.9650 (18)
Cu1—O2^i^	2.4646 (16)
Ca1—O3	2.3134 (16)
Ca1—O4^ii^	2.2818 (16)
Ca1—O5	2.3811 (16)

Symmetry codes: (i) 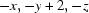; (ii) 
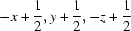
.

**Table 2:** Hydrogen-bond geometry (Å, °)

*D*—H⋯*A*	*D*—H	H⋯*A*	*D*⋯*A*	*D*—H⋯*A*
O1—H1*O*⋯O2	0.99	1.65	2.610 (2)	165
O5—H5*O*⋯O2^iii^	0.94	1.79	2.681 (2)	159

Symmetry code: (iii) 
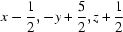
.

**Fig. 1.**
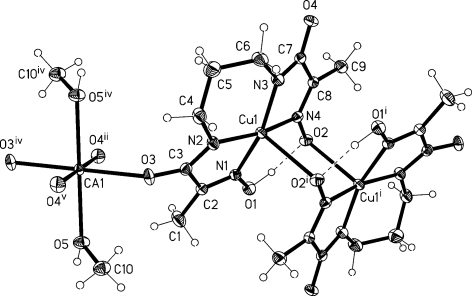
The structure of the title compound. Displacement ellipsoids are shown at the 50% probability level. Hydrogen bonds are indicated by dashed lines. [Symmetry codes: (i) -x, 2-y, -z; (ii) 1/2-x, 1/2+y, 1/2-z; (iv) -x, 2-y, 1-z; (v) -1/2+x, 3/2-y, 1/2+z.]

**Fig. 2.**
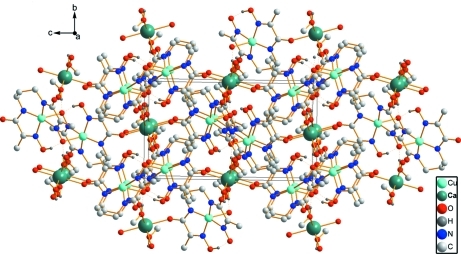
A packing diagram of the title compound. Hydrogen bonds are indicated by dashed lines. H atoms not involved in hydrogen bonding have been omitted for clarity.

## supplementary crystallographic information

### Comment

*N*,*N*'-bis(2-hydroxyiminopropionylpropane)-1,2-diamine and its homologues (Duda *et al.*, 1997; Fritsky *et al.*, 1999), tetradentate oxime-and-amide open-chain ligands, have been intensively studied during the past 15 years as efficient polychelate ligands forming stable complexes with nickel(II) and copper(II) ions. The presence of an additional strong donor amide function in the vicinity of the oxime group results in important increase of chelating efficiency. For example, amide derivatives of 2-hydroxyiminopropanoic acid were shown to act as highly efficient chelators with respect to copper(II), nickel(II) and aluminium(III) ions (Gumienna-Kontecka *et al.*, 2000; Onindo *et al.*, 1995; Sliva *et al.*, 1997a,b). Also, tetradentate oxime-and-amide open-chain ligands possess strong σ-donor capacity and thus have been successfully used for preparation of metal complexes with efficient stabilization of unusually high oxidation states of transition metal ions like Cu^III^ and Ni^III^ (Fritsky *et al.*, 1998; Fritsky *et al.*, 2006).

Earlier, the crystal and molecular structures of mononuclear anionic copper(II) complexes with *N*,*N*'-bis(2-hydroxyiminopropanoyl)propane-1,3-diamine (H_4_pap) of composition [Li(H_2_O)_4_][Cu(Hpap)].2H_2_O (Duda *et al.*, 1997) and PPh_4_[Cu(Hpap)].4.5H_2_O (Kanderal *et al.*, 2005) have been reported, as well as a series of modular cationic and anionic complex compounds containing [Cu(Hpap)]^-^ anions (Fritsky *et al.*, 2000). The present report describes the crystal structure of the title compound, a three-dimensional coordination polymer of composition [CaCu_2_(Hpap)_2_(CH_3_OH)_2_], featuring copper(II) complex anions connected by calcium ions.

The structure of the title compound is presented in Fig. 1. The ligand in the complex anion is coordinated in a tetradentate fashion forming three condensed chelate rings and being triply deprotonated. In the complex anion the Cu^II^ atom is situated in a distorted square-pyramidal geometry. The basal plane is defined by four N atoms belonging to the deprotonated amide and oxime groups of the Hpap ligand, which adopt a pseudo-macrocyclic conformation due to the presence of an intramolecular hydrogen bond uniting the *cis-*oximate O atoms. The apical position is occupied by the oxime O2 atom, and as a result, two neighboring Cu complex anions are united into a centrosymmetric [Cu_2_(Hpap)_2_]^2-^ dimer, with a Cu···Cu^i^ [symmetry code: (i) -x, 2-y, -z] separation of 4.164 (1) Å. Each dimeric unit connects four Ca atoms and each Ca links four dimeric [Cu_2_(Hpap)_2_]^2-^ units.

The basal plane of the Cu1 atom exhibits tetrahedral distortion with deviations of the N atoms from the mean plane defined by them by 0.025 (1) Å. Cu1 is displaced by 0.255 (1) Å from this plane in the direction of the apical O atom. The observed Cu—N distances (Table 1) are normal for the complexes with N-coordinated amide and oxime groups (Fritsky *et al.*, 1998; Fritsky *et al.*, 2006). A noticeable difference between Cu—N(amide) and Cu—N(oxime) distances is observed. The O1···O2 separation of the intramolecular hydrogen bond is equal to 2.610 (2) Å, which is close to the values reported for the analogous complexes with lithium and tetraphenylphosponium cations. The C═N, C═O, N—O and C—N bond lengths are typical for 2-hydroxyiminopropanoic acid and its amide derivatives (Fritsky, 1999; Mokhir *et al.*, 2002; Moroz *et al.*, 2008).

The Ca^II^ atom occupies a special position and is situated in moderately distorted octahedral environment (Fig. 1). The Ca—O bond distances are similar to the reported ones for six-coordinate calcium complexes (Wörl *et al.*,  2005). The axial bond length Ca1—O5 [2.381 (1) Å] are somewhat longer than the equatorial ones. The O—Ca—O anlges values are in the range 84.31 (6) to 95.69 (6)°. The coordination geometry of the Ca atom is formed by six O atoms belonging to two methanol molecules and four amide groups. Thus, each Ca atom unites four dimeric Cu complex anionic unit. These Ca—O bonds, together with the intermolecular O—H···O hydrogen bonds between the methanol OH group and oxime O2 atom (Table 2), lead to a three-dimensional framework (Fig. 2).

### Experimental

A solution of *N*,*N*'-bis(2-hydroxyiminopropanoyl)propane-1,3-diamine (0.244 g, 1 mmol) in 10 ml of methanol was heated to 323 K and added with stirring to a solution of copper(II) chloride dihydrate (0.170 g, 1 mmol) in water (5 ml). Then an aqueous solution of calcium hydrocarbonate (4 ml, 1 *M*) was added. The obtained mixture was stirred at 323 K for 10 min and then filtered. The filtrate was cooled, filtered and set aside for crystallization at room temperature. The resulting dark-red crystals formed within 12 h were separated by filtration, washed with water and air-dried (yield 78%). *N*,*N*'-bis(2-hydroxyiminopropanoyl)propane-1,3-diamine was prepared according to the reported procedure (Duda *et al.*, 1997).

### Refinement

O-bonded H atoms were located from a difference Fourier map and refined as riding atoms, with *U*_iso_ = 1.5*U*_eq_(O). H atoms of methyl and methylene groups were positioned geometrically and refined as riding atoms, with C—H = 0.99 (methylene) and 0.98 (methyl) Å, and *U*_iso_ = 1.2(1.5 for methyl)*U*_eq_(C).

### Crystal data

**Table:**

[CaCu_2_(C_9_H_13_N_4_O_4_)_2_(CH_4_O)_2_]	*F*(000) = 736
*M**_r_* = 713.71	*D*_x_ = 1.755 Mg m^−^^3^
Monoclinic, *P*2_1_/*n*	Mo *K*α radiation, λ = 0.71073 Å
Hall symbol: -P 2yn	Cell parameters from 3254 reflections
*a* = 10.0554 (4) Å	θ = 1.0–27.5°
*b* = 8.7794 (3) Å	µ = 1.83 mm^−^^1^
*c* = 15.4465 (7) Å	*T* = 120 K
β = 97.882 (2)°	Block, dark red
*V* = 1350.74 (9) Å^3^	0.28 × 0.24 × 0.13 mm
*Z* = 2	

### Data collection

**Table:**

Nonius KappaCCD diffractometer	3074 independent reflections
Radiation source: fine-focus sealed tube	2573 reflections with *I* > 2σ(*I*)
horizontally mounted graphite crystal	*R*_int_ = 0.035
Detector resolution: 9 pixels mm^-1^	θ_max_ = 27.5°, θ_min_ = 2.6°
φ and ω scans with κ offset	*h* = −11→13
Absorption correction: multi-scan (*SADABS*; Sheldrick, 1996)	*k* = −11→10
*T*_min_ = 0.622, *T*_max_ = 0.796	*l* = −20→19
8392 measured reflections	

### Refinement

**Table:**

Refinement on *F*^2^	Primary atom site location: structure-invariant direct methods
Least-squares matrix: full	Secondary atom site location: difference Fourier map
*R*[*F*^2^ > 2σ(*F*^2^)] = 0.030	Hydrogen site location: inferred from neighbouring sites
*wR*(*F*^2^) = 0.079	H-atom parameters constrained
*S* = 1.04	*w* = 1/[σ^2^(*F*_o_^2^) + (0.0351*P*)^2^ + 1.0009*P*] where *P* = (*F*_o_^2^ + 2*F*_c_^2^)/3
3074 reflections	(Δ/σ)_max_ = 0.002
192 parameters	Δρ_max_ = 1.11 e Å^−^^3^
0 restraints	Δρ_min_ = −0.56 e Å^−^^3^

### Fractional atomic coordinates and isotropic or equivalent isotropic displacement parameters (Å^2^)

**Table:**

	*x*	*y*	*z*	*U*_iso_*/*U*_eq_	
Cu1	0.07005 (3)	0.90813 (3)	0.122024 (17)	0.01389 (10)	
Ca1	0.0000	1.0000	0.5000	0.01418 (14)	
O1	−0.02176 (17)	1.23359 (18)	0.11376 (10)	0.0201 (4)	
H1O	0.0287	1.2169	0.0643	0.030*	
O2	0.11916 (16)	1.14306 (18)	−0.00651 (10)	0.0171 (3)	
O3	−0.05881 (17)	0.92919 (19)	0.35557 (10)	0.0213 (4)	
O4	0.38614 (16)	0.70750 (19)	0.04006 (11)	0.0210 (4)	
O5	−0.21320 (16)	1.12267 (19)	0.48180 (11)	0.0219 (4)	
H5O	−0.2529	1.2181	0.4868	0.033*	
N1	−0.01214 (18)	1.0977 (2)	0.15866 (12)	0.0146 (4)	
N2	0.01537 (19)	0.8358 (2)	0.23095 (12)	0.0173 (4)	
N3	0.1956 (2)	0.7474 (2)	0.10597 (12)	0.0180 (4)	
N4	0.15835 (18)	1.0068 (2)	0.03093 (12)	0.0144 (4)	
C1	−0.1226 (3)	1.2240 (3)	0.27158 (17)	0.0288 (6)	
H1A	−0.0570	1.3063	0.2843	0.043*	
H1B	−0.1542	1.1913	0.3259	0.043*	
H1C	−0.1989	1.2607	0.2305	0.043*	
C2	−0.0587 (2)	1.0934 (3)	0.23219 (15)	0.0169 (5)	
C3	−0.0343 (2)	0.9408 (3)	0.27836 (15)	0.0168 (5)	
C4	0.0562 (3)	0.6895 (3)	0.27209 (15)	0.0225 (5)	
H4A	−0.0109	0.6109	0.2505	0.027*	
H4B	0.0575	0.6985	0.3361	0.027*	
C5	0.1924 (3)	0.6394 (3)	0.25333 (17)	0.0294 (6)	
H5A	0.2165	0.5452	0.2872	0.035*	
H5B	0.2581	0.7185	0.2762	0.035*	
C6	0.2103 (3)	0.6087 (3)	0.15904 (17)	0.0251 (5)	
H6A	0.3006	0.5648	0.1570	0.030*	
H6B	0.1429	0.5329	0.1340	0.030*	
C7	0.2839 (2)	0.7824 (3)	0.05361 (14)	0.0163 (5)	
C8	0.2556 (2)	0.9315 (3)	0.00510 (14)	0.0158 (4)	
C9	0.3318 (2)	0.9800 (3)	−0.06579 (15)	0.0219 (5)	
H9A	0.2709	1.0303	−0.1120	0.033*	
H9B	0.3720	0.8905	−0.0899	0.033*	
H9C	0.4028	1.0511	−0.0423	0.033*	
C10	−0.3241 (2)	1.0385 (3)	0.43680 (17)	0.0251 (5)	
H10A	−0.2903	0.9503	0.4076	0.038*	
H10B	−0.3758	1.1041	0.3931	0.038*	
H10C	−0.3821	1.0035	0.4789	0.038*	

### Atomic displacement parameters (Å^2^)

**Table:**

	*U*^11^	*U*^22^	*U*^33^	*U*^12^	*U*^13^	*U*^23^
Cu1	0.01633 (15)	0.01304 (15)	0.01304 (14)	0.00299 (10)	0.00462 (10)	0.00150 (10)
Ca1	0.0144 (3)	0.0150 (3)	0.0135 (3)	−0.0026 (2)	0.0032 (2)	−0.0004 (2)
O1	0.0261 (9)	0.0151 (8)	0.0197 (8)	0.0043 (7)	0.0050 (7)	0.0032 (7)
O2	0.0183 (8)	0.0135 (7)	0.0195 (8)	0.0018 (6)	0.0021 (6)	0.0054 (6)
O3	0.0271 (9)	0.0250 (9)	0.0130 (8)	−0.0030 (7)	0.0071 (7)	−0.0015 (7)
O4	0.0192 (8)	0.0214 (8)	0.0231 (8)	0.0078 (7)	0.0053 (7)	−0.0014 (7)
O5	0.0176 (9)	0.0183 (8)	0.0293 (9)	0.0011 (6)	0.0012 (7)	−0.0042 (7)
N1	0.0148 (9)	0.0136 (9)	0.0153 (9)	0.0007 (7)	0.0015 (7)	0.0017 (7)
N2	0.0221 (10)	0.0158 (9)	0.0146 (9)	0.0019 (8)	0.0053 (8)	0.0026 (8)
N3	0.0225 (10)	0.0155 (9)	0.0167 (9)	0.0049 (8)	0.0057 (8)	0.0039 (8)
N4	0.0139 (9)	0.0140 (9)	0.0148 (9)	0.0009 (7)	0.0003 (7)	0.0009 (7)
C1	0.0398 (16)	0.0247 (13)	0.0239 (13)	0.0110 (11)	0.0120 (11)	−0.0002 (11)
C2	0.0155 (11)	0.0186 (11)	0.0165 (11)	0.0013 (9)	0.0020 (9)	−0.0017 (9)
C3	0.0148 (11)	0.0200 (11)	0.0151 (10)	−0.0021 (9)	0.0007 (8)	0.0000 (9)
C4	0.0309 (14)	0.0201 (12)	0.0175 (11)	0.0041 (10)	0.0071 (10)	0.0046 (10)
C5	0.0306 (14)	0.0299 (14)	0.0277 (14)	0.0073 (11)	0.0043 (11)	0.0093 (12)
C6	0.0306 (14)	0.0202 (12)	0.0257 (13)	0.0113 (10)	0.0082 (11)	0.0045 (10)
C7	0.0169 (11)	0.0165 (11)	0.0146 (10)	0.0030 (9)	−0.0008 (8)	−0.0016 (9)
C8	0.0148 (11)	0.0186 (11)	0.0138 (10)	0.0004 (9)	0.0010 (8)	−0.0012 (9)
C9	0.0195 (12)	0.0265 (13)	0.0209 (12)	0.0005 (10)	0.0065 (9)	0.0019 (10)
C10	0.0199 (12)	0.0237 (12)	0.0304 (13)	−0.0006 (10)	−0.0007 (10)	−0.0041 (11)

### Geometric parameters (Å, °)

**Table:**

Cu1—N1	1.9751 (18)	C1—C2	1.486 (3)
Cu1—N2	1.9469 (18)	C1—H1A	0.9800
Cu1—N3	1.9320 (19)	C1—H1B	0.9800
Cu1—N4	1.9650 (18)	C1—H1C	0.9800
Cu1—O2^i^	2.4646 (16)	C2—C3	1.522 (3)
Ca1—O3	2.3134 (16)	C4—C5	1.504 (4)
Ca1—O4^ii^	2.2818 (16)	C4—H4A	0.9900
Ca1—O5	2.3811 (16)	C4—H4B	0.9900
O1—N1	1.377 (2)	C5—C6	1.516 (4)
O1—H1O	0.9852	C5—H5A	0.9900
O2—N4	1.363 (2)	C5—H5B	0.9900
O3—C3	1.255 (3)	C6—H6A	0.9900
O4—C7	1.262 (3)	C6—H6B	0.9900
O5—C10	1.436 (3)	C7—C8	1.516 (3)
O5—H5O	0.9358	C8—C9	1.482 (3)
N1—C2	1.287 (3)	C9—H9A	0.9800
N2—C3	1.317 (3)	C9—H9B	0.9800
N2—C4	1.466 (3)	C9—H9C	0.9800
N3—C7	1.316 (3)	C10—H10A	0.9800
N3—C6	1.464 (3)	C10—H10B	0.9800
N4—C8	1.288 (3)	C10—H10C	0.9800
			
N3—Cu1—N2	98.02 (8)	H1B—C1—H1C	109.5
N3—Cu1—N4	82.10 (8)	N1—C2—C1	124.7 (2)
N2—Cu1—N4	166.31 (8)	N1—C2—C3	112.64 (19)
N3—Cu1—N1	163.47 (8)	C1—C2—C3	122.6 (2)
N2—Cu1—N1	81.29 (8)	O3—C3—N2	127.6 (2)
N4—Cu1—N1	94.69 (8)	O3—C3—C2	118.5 (2)
N3—Cu1—O2^i^	103.12 (7)	N2—C3—C2	113.87 (19)
N2—Cu1—O2^i^	106.54 (7)	N2—C4—C5	112.4 (2)
N4—Cu1—O2^i^	86.66 (6)	N2—C4—H4A	109.1
N1—Cu1—O2^i^	92.83 (7)	C5—C4—H4A	109.1
O4^iii^—Ca1—O4^ii^	180.00 (8)	N2—C4—H4B	109.1
O4^iii^—Ca1—O3^iv^	91.40 (6)	C5—C4—H4B	109.1
O4^ii^—Ca1—O3^iv^	88.60 (6)	H4A—C4—H4B	107.9
O4^iii^—Ca1—O3	88.60 (6)	C4—C5—C6	117.9 (2)
O4^ii^—Ca1—O3	91.40 (6)	C4—C5—H5A	107.8
O3^iv^—Ca1—O3	180.0	C6—C5—H5A	107.8
O4^iii^—Ca1—O5	85.18 (6)	C4—C5—H5B	107.8
O4^ii^—Ca1—O5	94.82 (6)	C6—C5—H5B	107.8
O3^iv^—Ca1—O5	95.69 (6)	H5A—C5—H5B	107.2
O3—Ca1—O5	84.31 (6)	N3—C6—C5	112.0 (2)
O4^iii^—Ca1—O5^iv^	94.82 (6)	N3—C6—H6A	109.2
O4^ii^—Ca1—O5^iv^	85.18 (6)	C5—C6—H6A	109.2
O3^iv^—Ca1—O5^iv^	84.31 (6)	N3—C6—H6B	109.2
O3—Ca1—O5^iv^	95.69 (6)	C5—C6—H6B	109.2
O5—Ca1—O5^iv^	180.0	H6A—C6—H6B	107.9
N1—O1—H1O	104.7	O4—C7—N3	127.8 (2)
C10—O5—H5O	100.9	O4—C7—C8	118.0 (2)
C2—N1—O1	117.47 (18)	N3—C7—C8	114.15 (19)
C2—N1—Cu1	116.42 (15)	N4—C8—C9	124.9 (2)
O1—N1—Cu1	126.09 (14)	N4—C8—C7	112.86 (19)
C3—N2—C4	118.48 (19)	C9—C8—C7	122.2 (2)
C3—N2—Cu1	115.18 (15)	C8—C9—H9A	109.5
C4—N2—Cu1	124.40 (15)	C8—C9—H9B	109.5
C7—N3—C6	120.9 (2)	H9A—C9—H9B	109.5
C7—N3—Cu1	114.57 (15)	C8—C9—H9C	109.5
C6—N3—Cu1	123.60 (15)	H9A—C9—H9C	109.5
C8—N4—O2	120.42 (18)	H9B—C9—H9C	109.5
C8—N4—Cu1	115.63 (15)	O5—C10—H10A	109.5
O2—N4—Cu1	123.91 (13)	O5—C10—H10B	109.5
C2—C1—H1A	109.5	H10A—C10—H10B	109.5
C2—C1—H1B	109.5	O5—C10—H10C	109.5
H1A—C1—H1B	109.5	H10A—C10—H10C	109.5
C2—C1—H1C	109.5	H10B—C10—H10C	109.5
H1A—C1—H1C	109.5		
			
N3—Cu1—N1—C2	−87.3 (3)	N2—Cu1—N4—O2	−93.4 (3)
N2—Cu1—N1—C2	1.52 (17)	N1—Cu1—N4—O2	−21.15 (16)
N4—Cu1—N1—C2	−165.30 (17)	O2^i^—Cu1—N4—O2	71.42 (16)
O2^i^—Cu1—N1—C2	107.82 (17)	Cu1^i^—Cu1—N4—O2	50.60 (13)
Cu1^i^—Cu1—N1—C2	150.40 (17)	O1—N1—C2—C1	0.4 (3)
N3—Cu1—N1—O1	91.4 (3)	Cu1—N1—C2—C1	179.3 (2)
N2—Cu1—N1—O1	−179.78 (18)	O1—N1—C2—C3	−176.28 (17)
N4—Cu1—N1—O1	13.41 (17)	Cu1—N1—C2—C3	2.5 (2)
O2^i^—Cu1—N1—O1	−73.47 (17)	C4—N2—C3—O3	−4.2 (4)
Cu1^i^—Cu1—N1—O1	−30.90 (16)	Cu1—N2—C3—O3	−169.08 (19)
N3—Cu1—N2—C3	157.38 (17)	C4—N2—C3—C2	173.6 (2)
N4—Cu1—N2—C3	67.8 (4)	Cu1—N2—C3—C2	8.8 (2)
N1—Cu1—N2—C3	−5.93 (16)	N1—C2—C3—O3	170.6 (2)
O2^i^—Cu1—N2—C3	−96.30 (17)	C1—C2—C3—O3	−6.2 (3)
Cu1^i^—Cu1—N2—C3	−65.8 (2)	N1—C2—C3—N2	−7.4 (3)
N3—Cu1—N2—C4	−6.4 (2)	C1—C2—C3—N2	175.8 (2)
N4—Cu1—N2—C4	−96.0 (4)	C3—N2—C4—C5	−133.0 (2)
N1—Cu1—N2—C4	−169.8 (2)	Cu1—N2—C4—C5	30.3 (3)
O2^i^—Cu1—N2—C4	99.87 (19)	N2—C4—C5—C6	−62.6 (3)
Cu1^i^—Cu1—N2—C4	130.39 (16)	C7—N3—C6—C5	132.3 (2)
N2—Cu1—N3—C7	−159.43 (17)	Cu1—N3—C6—C5	−35.9 (3)
N4—Cu1—N3—C7	6.74 (16)	C4—C5—C6—N3	65.8 (3)
N1—Cu1—N3—C7	−73.0 (3)	Ca1^v^—O4—C7—N3	57.7 (5)
O2^i^—Cu1—N3—C7	91.42 (17)	Ca1^v^—O4—C7—C8	−122.4 (3)
Cu1^i^—Cu1—N3—C7	45.67 (17)	C6—N3—C7—O4	1.4 (4)
N2—Cu1—N3—C6	9.4 (2)	Cu1—N3—C7—O4	170.55 (19)
N4—Cu1—N3—C6	175.6 (2)	C6—N3—C7—C8	−178.6 (2)
N1—Cu1—N3—C6	95.8 (3)	Cu1—N3—C7—C8	−9.4 (2)
O2^i^—Cu1—N3—C6	−99.71 (19)	O2—N4—C8—C9	−0.9 (3)
Cu1^i^—Cu1—N3—C6	−145.46 (18)	Cu1—N4—C8—C9	176.75 (18)
N3—Cu1—N4—C8	−2.44 (16)	O2—N4—C8—C7	−179.45 (17)
N2—Cu1—N4—C8	89.0 (4)	Cu1—N4—C8—C7	−1.8 (2)
N1—Cu1—N4—C8	161.24 (16)	O4—C7—C8—N4	−172.59 (19)
O2^i^—Cu1—N4—C8	−106.19 (16)	N3—C7—C8—N4	7.3 (3)
Cu1^i^—Cu1—N4—C8	−127.01 (18)	O4—C7—C8—C9	8.9 (3)
N3—Cu1—N4—O2	175.17 (17)	N3—C7—C8—C9	−171.2 (2)

Symmetry codes: (i) −*x*, −*y*+2, −*z*; (ii) −*x*+1/2, *y*+1/2, −*z*+1/2; (iii) *x*−1/2, −*y*+3/2, *z*+1/2; (iv) −*x*, −*y*+2, −*z*+1; (v) −*x*+1/2, *y*−1/2, −*z*+1/2.

### Hydrogen-bond geometry (Å, °)

**Table:**

*D*—H···*A*	*D*—H	H···*A*	*D*···*A*	*D*—H···*A*
O1—H1O···O2	0.99	1.65	2.610 (2)	165
O5—H5O···O2^vi^	0.94	1.79	2.681 (2)	159

Symmetry codes: (vi) *x*−1/2, −*y*+5/2, *z*+1/2.
